# LncRNA FALEC increases the proliferation, migration and drug resistance of cholangiocarcinoma through competitive regulation of miR-20a-5p/SHOC2 axis

**DOI:** 10.18632/aging.204709

**Published:** 2023-05-09

**Authors:** Haiming Du, Senlin Hou, Lichao Zhang, Chao Liu, Tingting Yu, Wei Zhang

**Affiliations:** 1 The Biliopancreatic Endoscopic Surgery Department, The Second Hospital of Hebei Medical University, Shijiazhuang, Hebei Province, China; 2 Department of Anesthesiology, The Second Hospital of Hebei Medical University, Shijiazhuang, Hebei Province, China

**Keywords:** cholangiocarcinoma, lncRNA FALEC, miR-20a-5p, SHOC2, ERK1/2

## Abstract

Background: LncRNA is an important regulatory factor in the human genome. We aim to explore the roles of LncFALEC and miR-20a-5p/SHOC2 axis on the proliferation, migration, and Fluorouracil (5-FU) resistance of cholangiocarcinoma (CCA).

Methods: In this study, the expression of FALEC and miR-20a-5p in CCA tissues and cell lines (HuCCT1, QBC939, and Huh-28) was detected by RT-qPCR. The FALEC in 5-FU-resistant CCA cell lines (QBC939-R, Huh-28-R) was knocked down to evaluate its effects on cell proliferation, migration, invasion, and drug resistance.

Results: Our analysis showed that compared with the adjacent non-tumor tissues, FALEC was significantly higher in the CCA tissues and even higher in the samples from 5-FU-resistant patients. Knockdown FALEC increased the sensitivity of 5-FU and decreased migration and invasion of CCA cells. Dual luciferase reporter confirmed that FALEC sponges miR-20a-5p and down-regulated its expression. Moreover, SHOC2 leucine-rich repeat scaffold protein (SHOC2) was the target gene of miR-20a-5p. We found overexpression of FALEC (FALEC-OE) increased resistance of CCA cells to 5-FU significantly, which might contribute to increased SHOC2 expression and activation of the ERK1/2 signaling pathway.

Conclusions: In summary, our study revealed that down-regulation of FALEC could inhibit the proliferation, migration, and invasion of CCA cells *in vitro* by regulating the miR-20a-5p/SHOC2 axis and participating in 5-FU resistance by mediating the ERK1/2 signaling pathway.

## INTRODUCTION

Cholangiocarcinoma (CCA) is one of the most common malignant tumors, with a higher incidence and mortality rate. Recent epidemiological investigation showed that the mortality rate of CCA still increased in many countries and regions [1]. The main treatment strategies for CCA include surgical resection, chemotherapy, radiotherapy, and targeted therapies [2]. However, many patients miss the best opportunity for surgery because of the late detection of tumors. Achieving the goal of early detection and early treatment of CCA requires continuous research on the process of tumor pathogenesis and progression. LncRNA refers to a non-coding RNA with a length of >200 nucleotides and has a non-protein-coding function [3]. As an important regulatory factor in the human genome, lncRNA regulates histone modification, DNA methylation, or chromosome reconstruction through epigenetic regulation, transcriptional or post-transcriptional regulation, and other mechanisms to silence or activate genes, and then dynamically control disease-related gene changes and some important biological processes [4]. The abnormal expression of lncRNA can participate in both tumor inhibition and carcinogenesis [5]. Currently, many lncRNAs have been confirmed to be abnormally expressed in various organ tumors. They interact with microRNAs via multiple mechanisms regulating gene expression [5].

Focally amplified lncRNA on chromosome 1 (FALEC) is a novel lncRNA located in a focal amplicon on chromosome 1q21.2. The local amplification of FALEC has been identified as an oncogenic property in a variety of human cancers, including endometrial cancer [6], tongue squamous cell carcinoma [7], and colorectal cancer [8]. Wu et al. [9] found that the expression of FALEC was significantly upregulated in gastric cancer tissue compared to paired non-tumor tissues, thereby possibly involved in promoting the migration and invasion ability of gastric cancer cells. MicroRNAs (miRNAs) are highly conserved short single-stranded non-coding RNAs consisting of 18 to 22 nucleotides. They are involved in a variety of biological functions by targeting and binding mRNA and participating in gene silencing, translation, and suppression [10, 11]. The previous studies reported that miR-20a-5p, act as an onco-miRNA or anti-cancer gene, promotes or inhibits cancer progress dependent with the type of cancer [12]. Jones et al. [13] indicated that SHOC2, a scaffold protein, participates tumor progression and induce drug resistance. Few reports mentioned roles of above genes in CCA. In the current study, we aimed to explore the role of these signaling molecules in the CCA progression, and their further detailed effect in drug resistance.

## MATERIALS AND METHODS

### Samples and cell lines

All clinical CCA samples were obtained from the second hospital of Hebei medical university. Twenty pathologically confirmed CCA tissues and adjacent non-tumor tissues with complete medical record were finally selected. Samples were immediately frozen in liquid nitrogen after surgery and then stored at -80° C refrigerator. Detailed criteria including patients did not receive chemotherapy or radiotherapy before surgery; patients received Fluorouracil (5-FU) as the first-line chemotherapy drug after surgery at least 6 months. Written informed consent was obtained for using and analyzing their samples. This study was approved by the Ethics Committee of the second hospital of Hebei medical university. Their sensitivity to chemotherapy was judged as to achieve complete clinical remission or no recurrence within 6 months after chemotherapy. The chemotherapy resistance is considered as the continuous progress of the disease during chemotherapy treatment or the recurrence 6 months after chemotherapy.

Human intrahepatic biliary epithelial cell (HIBEC), CCA cell lines including HuCCT1, QBC939, and Huh-28 were all obtained from Shanghai Cell Bank of Chinese Academy of Sciences. HIBEC and the three CCA cell lines were cultured in RPMI-1640 medium (11875168, Thermo Fisher Scientific, USA) with 10% FBS (11875085, Thermo Fisher) supplemented with 100 U/mL penicillin and 100 μg/mL streptomycin in a humidified cell incubator with 5% CO_2_ at 37° C.

### Construction of cell line resistant to 5-FU and transfection

Cells resistant to 5-FU (858471, Sigma, MO, USA; dissolved in DMSO) were established and selected through increasing concentrations of 5-FU in cultured medium as a previously reported method [14]. Briefly, CCA cells were firstly cultured in the medium containing 0.1 μg/ml 5-FU for 2 days, then replaced with 5-FU free medium and cultured for another 2 days; Subsequently, the dose of 5-FU is continuously increased to 1, 10, 20, 40 and 80 μg/ml. Cells stable passaged in medium containing 80 μg/ml 5-FU were selected and cultured in the medium supplemented with 5-FU to maintain their resistance. The 5-FU resistant cells were termed as QBC939-R and Huh-28-R respectively.

The human miR-20a-5p mimic, negative control mimic (miR10000075-1-5 and miR1N0000001-1-5), as well as interfering sequences of FALEC (lnc3180628030057) and SHOC2 (siG000008036B-1-5), were acquired from RiboBio Inc., (Guangzhou, China). Overexpression plasmids of FALEC (FALEC OE) were constructed by pCMV3-N-GFPSpark® carrier (Sino Biological Inc., Beijing, China). All sequences are protected by patents. All functional experiments were performed 48 hours after the transient transfection. Briefly, cells were added in triplicate to a 96-well microplate with concentration of 1 × 10^4^ cells/well. Transfection were performed with Lipofectamine 2000 reagent (11668019, Invitrogen, CA, USA) after cells were cultured overnight. Prepared mimic, NC mimic or duplexes of small interfering RNA (siRNA, 0.6 μg) were diluted by Opti-MEM® medium and incubated with diluted lipofectamine® 2000 Reagent (1:1 ratio) according to manufacturer’s instruction, cells were harvested 48 hours after transfection. qRT-PCR was applied to validate the transfection efficiency.

### Quantitative real-time reverse transcription (qRT-PCR) analysis

Total RNA was isolated from 20 clinical samples and CCA cells using TRIzol reagent (R1100 Solarbio Life Science, Beijing, China) following the manufacturer’s protocol. Sixty hundred nanogram total RNA per sample was used. PrimeScript™ RT reagent Kit with gDNA Eraser (RR047A, Takara Bio, Tokyo, Japan) was used for complementary DNA (cDNA) synthesis. After cDNA synthesis, SYBR Premix Ex Taq™ II (RR820A, Takara Bio) was adopted to perform qRT-PCR using the LightCycler 480 II Instrument (Roche Molecular Systems, Inc., CA, USA). The total reaction volume was 10 μl, including 5 μl of 2 x SYBR Green PCR buffer, 0.4 μl of forwarding primer (10 mM), 0.4 μl of reverse primer (10 mM), 0.2 μl of ROX Reference Dye II, 3.5 μl of ddH2O, and 15 ng of cDNA. PCR reaction conditions were set as: 95° C 30s; 95° C 5s; 60° C 30s; 40 cycles, and 65° C 15s. Relative quantification of RNA expression was calculated using the 2^−ΔΔCt^ method. U6 and GADPH were adopted as internal reference gene.

Used primers were: miR-20a-5 (forward, 5′ -TAAAGTGCTTATAGTGCAGGTAG-3′; reverse, 5'-TGGTGTCGTGGAGTCG-3'). U6 (forward, 5’- CTCGCTTCGGCAGCACAT-3’; reverse, 5’- TTTGCGTGTCATCCTTGCG-3’). FALEC: forward, 5’-CCTGGCCAAGAAGCTCATAC-3’ and reverse, 5’-TGAGGACACCGACTACTGAGAA3’. SHOC2 (forward, 5'- TCAGTGGTGTATAGGCTGGATTCT-3'; reverse, 5'-GCTACATCCAGCGTAATGAGGT-3’). GADPH (forward, 5’-TCAAGGCTGAGAACGGGAAG-3’; Reverse: 5’-CGCCCCACTTGATTTTGGAG-3’).

### Cell proliferation assay

Cell proliferation was measured by CCK-8 method in 2 5-FU resistant cell lines, QBC939-R and Huh-28-R. Cells (5×10^3^ cells/well) in logarithmic growth stage were loaded in triplicate to 96-well plate. Cells were transfected with the following sequence: si-FALEC, FALAC OE, miR-20a-5p mimic, si-SHOC2 and appropriate negative control sequences following the above mentioned method. The cells were cultured for 24, 48 and 72 h, respectively, and 10 μL CCK-8 reagent (CA1210, Solarbio Life Science, Beijing, China) was added to each well and incubated for another 2 hours before measuring cell proliferation. OD value at 450 nm of each well was measured by iMark microplate reader (Bio-Rad, CA, USA). Cell viability (%) = (OD value of experimental group - OD value of blank control group) / OD value of blank control group × 100%.

### Colony formation assay

Colony formation was evaluated in a 12-well plate. Briefly, 1000 μL medium containing 3×10^2^ cell/mL of resuspended QBC939-R and Huh-28-R cells, transfected with si-FALEC or negative control sequence, were loaded in a plate. Cells were cultured for 14 days, and the culture medium was removed. After washed by PBS, cells were fixed with 4% paraformaldehyde for 30 min and stained with 0.05% crystal violet for 30 min. The number of colonies were counted (>20 cells were recorded record as one clone) and images were captured.

### Wound healing assay

The wound healing assay was performed as described in previous publication [15, 16]. Briefly, a total of 5×10^4^ cells were inoculated in a 6-well plate and ensure cells were evenly distributed. After cultured in an incubator for 4 hours, a sterilized 20 μl pipette tip was used to generate wounding across the cell monolayer. The debris of cells was washed with PBS and cultured for another 48 hours. The width of scratches in QBC939-R and Huh-28-R cells with or without knocking down FALEC were observed under a microscope at 0, 24, and 48 hours. The images were captured, and scratch width was calculated by ImageJ software (NIH, USA).

### Transwell assay

The Transwell assay was used to detect migration and invasion ability of CCA cell after knocking down FALEC. Matrigel (1:20) was added to the upper chamber of transwell inserts (3428, Corning Life Science, USA) in 6-well plate. After serum removal by starvation, 200 μL cell (5×10^4^ cells/ml) was added to the upper layer of transwell chamber 48 hours after transfection. A total of 600 μL of medium containing 10% FBS was added to the lower chamber. The plate was cultured in 37° C for 12 hours, the non-invasive cells in the upper chamber were wiped with cotton swabs and invasive cells were stained with 0.1% crystal violet. Five fields were randomly selected under a light microscope to observe and count the number of invasive cells.

### Dual-luciferase reporter assay

We obtained the cDNA clone of FALEC from NCBI (Gene ID: 100874054). The plasmid (C8021, psiCHECK™-2 Vectors, Promega) containing the full length wild-type (WT) or mutant (Mut) FALEC miRNA response elements (MREs) of miR-20a-5p were constructed. The miR-20a-5p mimic or NC mimic were co-transfected to QBC939 cells in a 96-well plate by lipofectamine® 2000 reagent. After incubated for 48 hours, the activities of firefly and Renilla luciferase were measured using the Dual Luciferase Reporter Assay Kit (E1910, Promega) following provided protocols, the firefly luciferase activity was normalized to the Renilla luciferase activity. The same methods were also used for testing the direct binding between miR-201-5p and SHOC2, and the plasmid (psiCHECK™-2 Vectors) containing the full length wild-type (WT) or mutant (Mut) miRNA response elements (MREs) of SHOC2 were constructed.

### Western blot analysis

Western blot was performed as per previous publications [17–19]. Cells were lysed by 200 μL RIPA lysis buffer (R0010, Solarbio) and then centrifuged with 12000 rpm for 4 minutes at 4° C. The supernatant was obtained to separate proteins. An BCA Protein Assay Kit (PC0020, Solarbio) was used to detect the concentration of total proteins.

Total proteins of the same concentration (30 μg) were loaded in 10% gel of SDS-PAGE. The separated proteins were transferred to nitrocellulose membranes (Millipore, MA, USA) and incubated overnight together with primary antibodies, including anti-SHOC2 (1:1000 dilution, ab229805, Abcam, MA, USA), anti-ERK1/2 (1:1000 dilution, ab184699, Abcam), anti-pERK1/2 (1:1000 dilution, ab65142, Abcam) and anti-β-actin (1:1000 dilution, ab8226, Abcam). Then, the membranes and goat antirabbit IgG H&L (HRP) secondary antibody (ab205718, Abcam) were incubated at room temperature for 1 hour. Then the membranes were immersed in 200 μL Immobilon Western Chemiluminescent HRP substrate (WBKLS0100, Millipore) and protein signals were recorded by the Bio-Rad ChemoDox XRS System (Bio-Rad Laboratories, CA, USA).

### Statistical analysis

The data obtained were statistically analyzed by GraphPad Prism 8.0 (GraphPad Software, Inc., CA, USA). Results were collected from three independent experiments and expressed as mean ± SD. Student's t-test or one-way analysis of variance was used for analysis of significant differences. A two-sided p < 0.05 was considered statistically significant.

### Data availability

The dataset of this study is available from the corresponding author upon reasonable request.

## RESULTS

### Lnc FALEC expression is upregulated in CCA samples and cell lines

**Figure 1:**
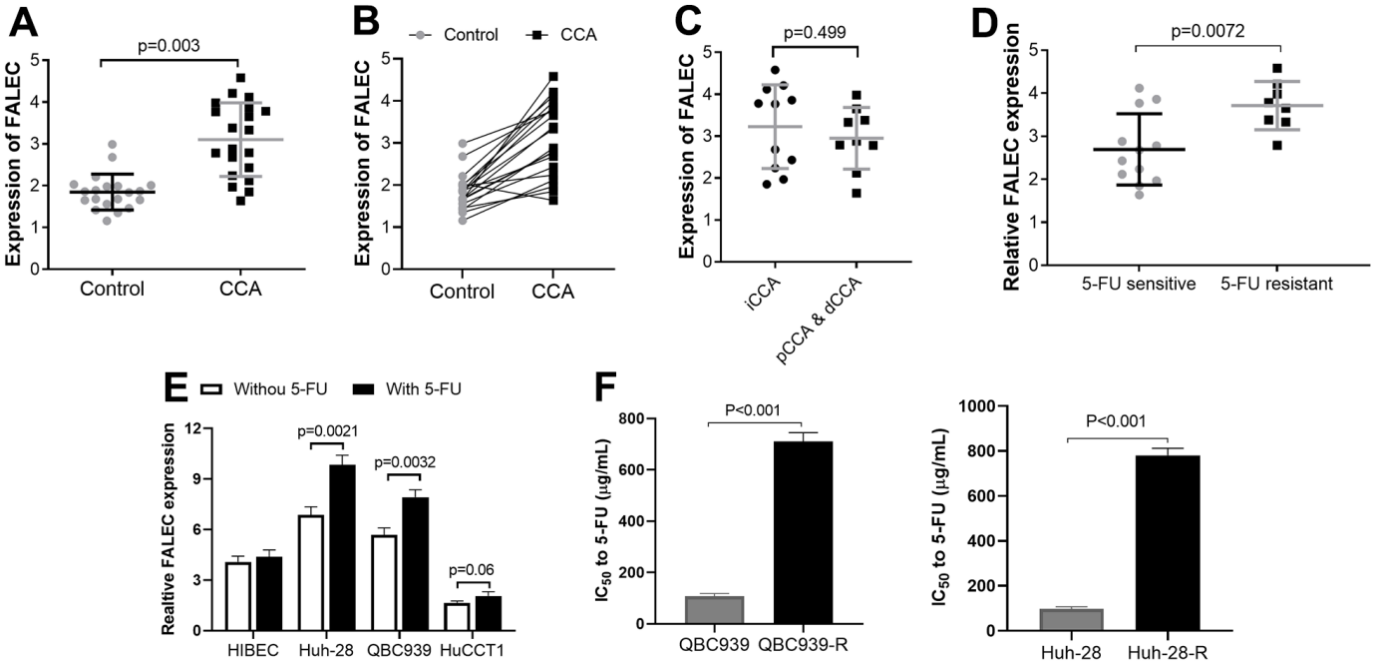
**The upregulated FALEC expression in cholangiocarcinoma (CCA) samples (n=20) and cell lines, the expression was analyzed by RT-PCR.** (**A**, **B**) The mRNA levels of FALEC were up-regulated in CCA samples compared to adjacent normal tissues; (**C**) The mRNA levels of FALEC were not different in in CCA at different anatomical locations. (**D**) The mRNA levels of FALEC are significantly higher in patients who were resistant to 5-FU, compared to 5-FU sensitive patients; (**E**) The expressions of FALEC in 3 CCA cell lines were measured with and without 5-FU stimulation (100 μg/ml) compared with HIBEC cells. QBC939, and Huh-28 cell lines have higher FALEC expression and selected for further analysis; (**F**) IC_50_ of normally cultured CCA cells and their corresponding 5-FU resistant cells.

A total of twenty CCA tumor tissues and their match adjacent non-tumor tissues were involved successfully. The 20 patients included 15 males and 5 females, with an average age of 61.9±6.19 years; 11 had intrahepatic cholangiocarcinoma (iCCA) and 9 had perihilar cholangiocarcinoma (pCCA) and distal cholangiocarcinoma (dCCA). Pathological examination results showed 12 were in stage I to II, 8 were in stage III to IV. Metastasis was confirmed in 7 patients. qRT-PCR analysis was used to analyze FALEC expression. Compared with adjacent non-tumor control, FALEC increased in CCA samples (Figure 1A, 1B), however, expression of FALEC has no difference in CCA at different anatomical locations (Figure 1C). In patients who were resistant to 5-FU, FALEC was significantly higher than in patients who were sensitive to 5-FU (Figure 1D). The expressions of FALEC in HIBEC and another 3 CCA cell lines were measured with and without 5-FU stimulation (100 μg/ml). We found its expressions in 3 CCA cell lines were all increased compared with HIBEC, and CCA cell line QBC939 and Huh-28 had higher FALEC expression (Figure 1E). These two CCA cells were induced to establish stable 5-FU resistant cell lines and were used for further analysis, and their increased half maximum inhibitory concentration (IC_50_) was shown in Figure 1F. Significantly increased IC_50_ to 5-FU also confirmed the successful establishment of drug resistant CCA cells.

### Knocking down FALEC increases the sensitivity of 5-FU, decreases proliferation, migration and invasion of CCA cells

**Figure 2:**
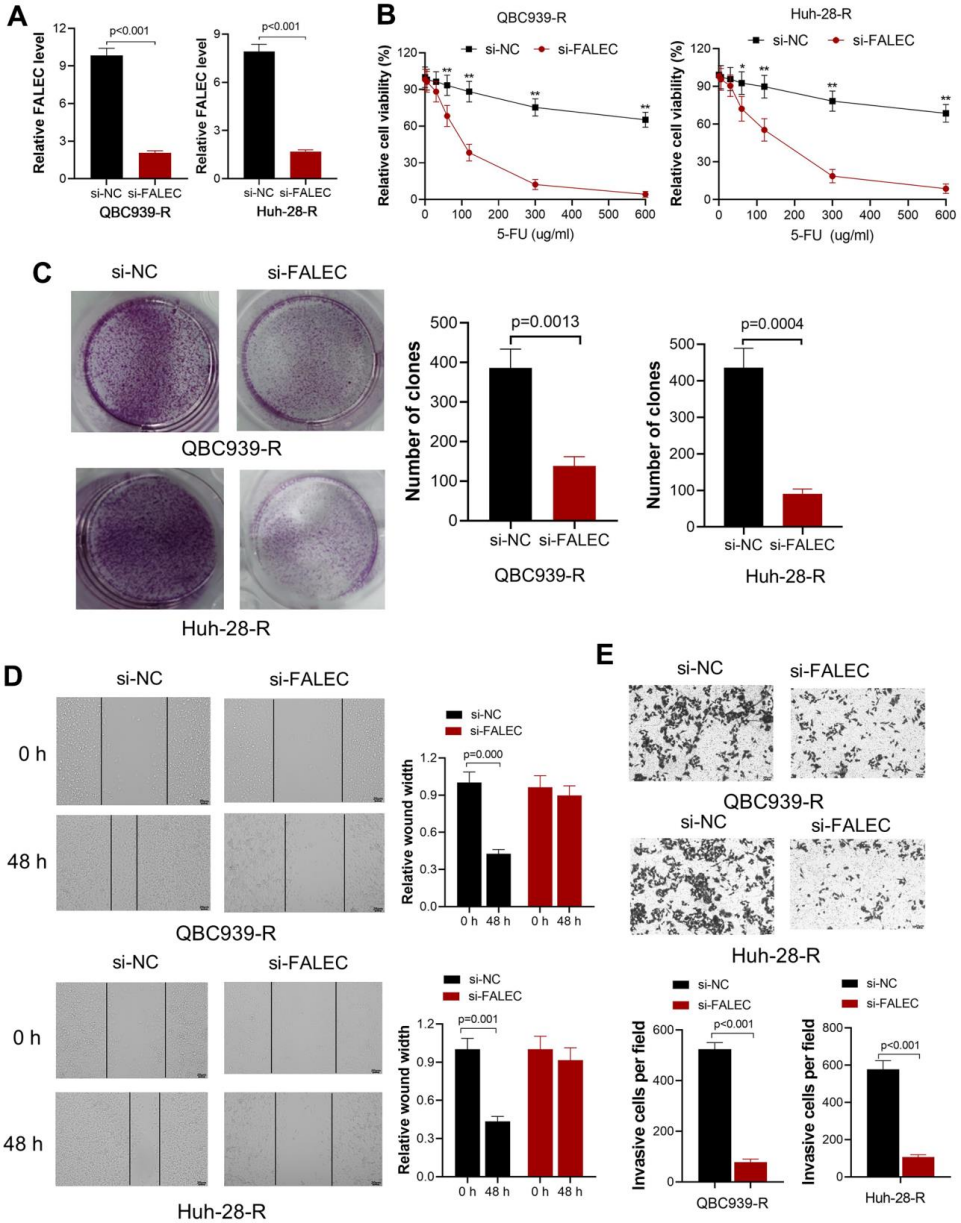
**Knockdown FALEC increase the sensitivity of 5-FU resistant CCA cells.** (**A**) FALEC level was significantly reduced in 5-FU resistant cell lines (QBC939-R and Huh-28-R) after transfected by si-FALEC. (**B**) Cell viability of QBC939-R and Huh-28-R decreased after transfected by si-FALEC; ^*^, p<0.05, ^**^, p<0.01, compared with negative control sequence (si-NC); (**C**) The proliferation ability of cells was measured by clone formation assay. Knocking down FALEC decreases the clone formation of QBC939-R and Huh-28-R significantly; (**D**) The migration ability of QBC939-R and Huh-28-R cells were measured by scratch method. Knocking down FALEC decreased the migration ability of both cell lines; (**E**) The invasion ability of QBC939-R and Huh-28-R cells decreased after knocking down FALEC.

Aims to investigate biological effect of FALEC in CCA, a si-FALEC with the highest transfection efficacy was transfected into the two 5-FU resistant cell lines, QBC939-R and Huh-28-R. By RT-qPCR, the significantly decreased FALEC in both cell lines was confirmed (Figure 2A). The sensitivity of QBC939-R and Huh-28-R stimulated by different 5-FU concentrations were measured after cells were cultured in 5-FU for 48 hours. We observed decreased cell viability after knocking down the FALEC (Figure 2B) in both 5-FU resistant cell lines. The proliferation ability was measured by clone formation assay. Results also showed that knocking down the FALEC significantly decreased the proliferation of both cell lines (Figure 2C). Moreover, migration analyzed by wound healing showed the migration of both cells was inhibited significantly after knocking down FALEC (Figure 2D). The invasion measured by transwell assay also confirmed knocking down of FALEC decreased the invasion ability of in 5-FU resistant CCA cells (Figure 2E).

### FALEC suppresses miR-20a-5p expression in CCA cells

**Figure 3:**
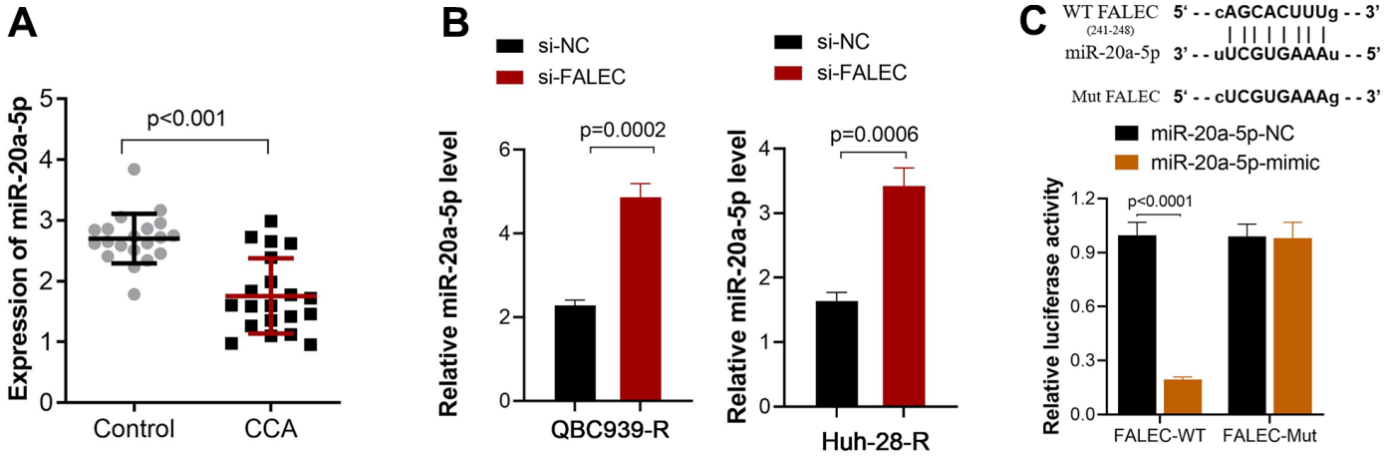
**FALEC suppresses miR-20a-5p expression by direct interaction.** (**A**) Decreased miR-20a-5p expression in CCA tissue when compared to adjacent non-tumor tissues, and the expression was analyzed by RT-PCR; (**B**) The expression of miR-20a-5p increased after FALEC was blocked in QBC939-R and Huh-28-R cells; (**C**) The binding site between FALEC and miR-20a-5p was verified by dual luciferase reporter assay. The luciferase activity of the wild-type FALEC sequence (WT-FALEC) decreased significantly after the mimic sequence of miR-20a-5p was transfected into cells.

FALEC is a non-coding RNA, and bioinformatics analysis (ENCORI) showed that the potential binding proteins of FALEC are not involved in the drug resistance process. This means FALEC may exert its function by targeting miRNA. Bioinformatics analysis showed that miR-20a-5p is one potential binding target of FALEC. The expression analysis showed that miR-20a-5p in CCA tumor tissues remarkably decreased compared to adjacent non-tumor tissues (P<0.001, Figure 3A). Further, the expression of miR-20a-5p in QBC939-R and Huh-28-R cells increased after FALEC was knocked down by its interfering sequence (Figure 3B). The luciferase reporter results showed that luciferase activity of the wild-type FALEC sequence (WT-FALEC) decreased significantly after the mimic sequence of miR-20a-5p was transfected into cells, but not mutant sequence (Figure 3C).

### miR-20a-5p downregulates the expression of SHOC2 in CCA cells via direct interaction

**Figure 4:**
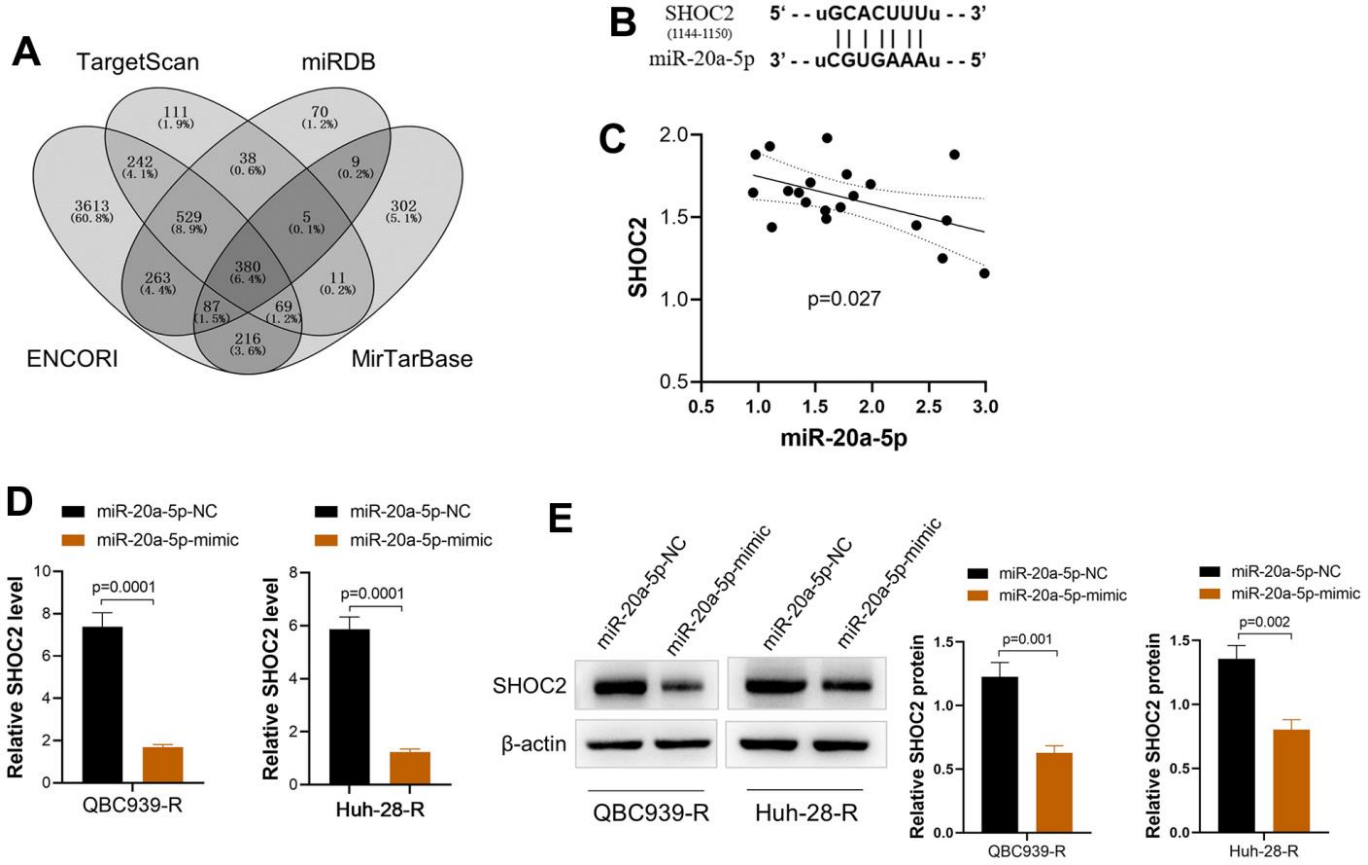
**miR-20a-5p downregulates SHOC2 in 5-FU resistant CCA cells via direct binding.** (**A**) The potential target gene of miR-20a-5p predicted via online tools (ENCORI, Target Scan, miRDB, and MirTarBase) and drawn by Venny online analysis software; (**B**) The predicted binding site of SHOC2 and miR-20a-5p; (**C**) The mRNA expression of SHOC2 was negatively correlated with the miR-20a-5p in CCA tissues; (**D**, **E**) upregulation of miR-20a-5p by its mimic decreased the expression of SHOC2 both in mRNA (**D**) and protein levels (**E**) of QBC939-R and Huh-28-R cells.

We screened the potential targeted functional gene of miR-20a-5p via online tools (ENCORI, Target Scan, miRDB, and MirTarBase). A total of 380 common genes were obtained via Venny online analysis software (Figure 4A). The involved pathway of miR-20a-5p was further analyzed by DIANA-miRPath [20] via KEGG pathway analysis and the results demonstrated MAPK pathway was involved in the function of miR-20a-5p. SHOC2 Leucine-Rich Repeat Scaffold Protein (SHOC2), a gene of MAPK pathway [13], with a conserved binding site with miR-20a-5p was finally identified as a potential target of miR-20a-5p (Figure 4B). Correlation analysis showed that mRNA expression of SHOC2 was negatively correlated with the miR-20a-5p in CCA tissues (Figure 4C). In addition, upregulation of miR-20a-5p decreased the expression of SHOC2 both in mRNA and protein levels in QBC939-R and Huh-28-R cells (Figure 4D, 4E).

### FALEC increased the 5-FU resistance of CCA cells by enhancing SHOC2 expression via decreasing miR-20a-5p

**Figure 5:**
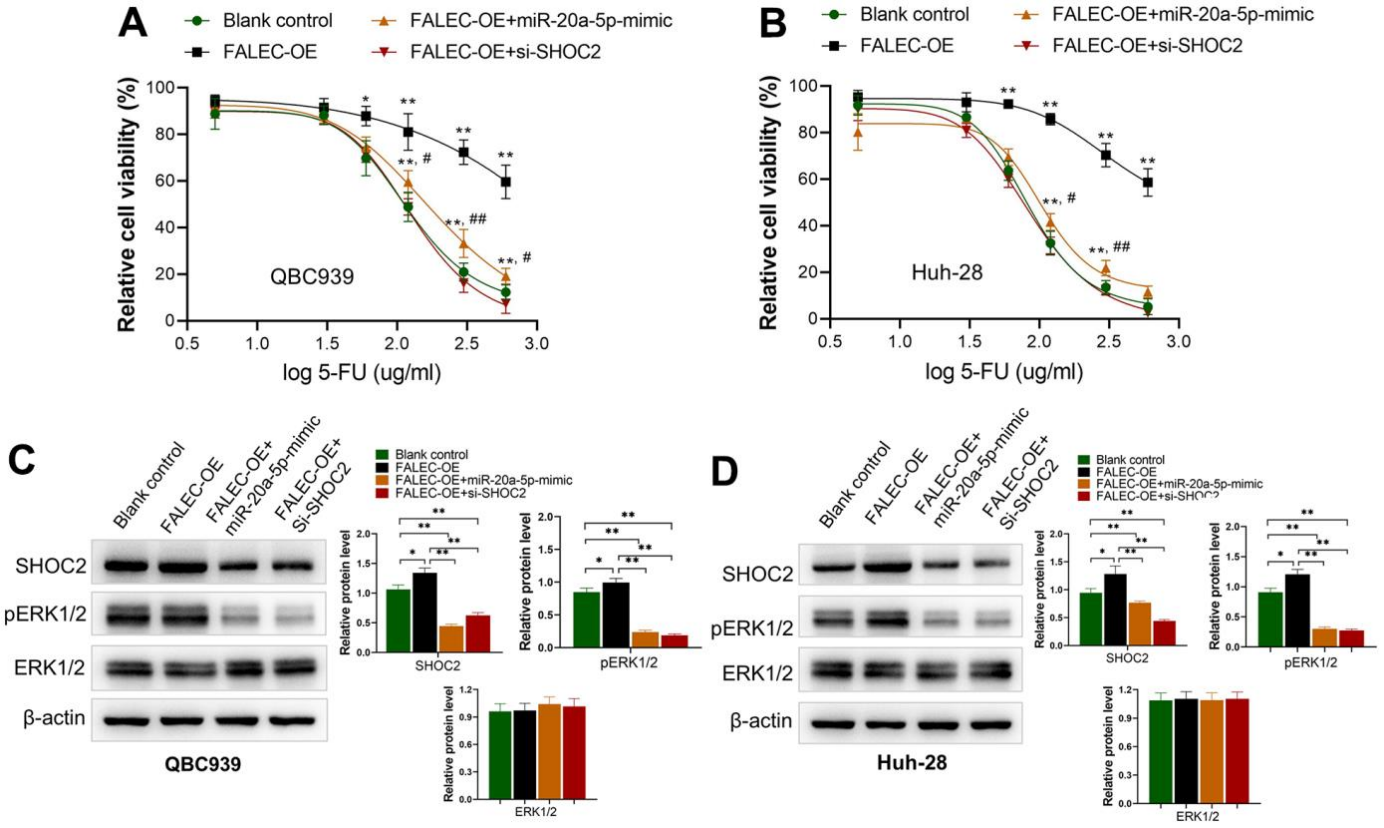
**FALEC increased the 5-FU resistance of CCA cells by miR-20a-5p/SHOC2 axis.** (**A**, **B**) Overexpression of FALEC (FALEC-OE) in normally cultured QBC939 and Huh-28 cells increased their resistance to 5-FU significantly in both cells; ^*^, p<0.05, ^**^, p<0.01, compared with blank control; ^#^, p<0.05, ^##^, p<0.01, compared with FALEC-OE; (**C**, **D**) protein expression of SHOC2 and its downstream activator protein pERK1/2 increased after enhancing FALEC expression (FALEC-OE) in both cell lines. Upregulation of miR-20a-5p or knockdown SHOC2 (si-SHOC2) abolished the enhancing effect to SHOC2, and pERK1/2 caused by FALEC.

To clarify whether FALEC increased 5-FU resistance was dependent with miR-20q-5p/Shoc2 axis, FALEC OE plasmid vector was transfected into 5-FU unstimulated CCA cells. Results demonstrated level of FALEC (FALEC-OE) in QBC939 and Huh-28 cells increased their resistance to 5-FU significantly in both cells (Figure 5A, 5B). The sensitivity of both cell lines was partially restored after the miR-20a-5p mimics was co-transfected. On the other hand, knocking down of SHOC2 by si-SHOC2 also abolished 5-FU resistance of QBC939 and Huh-28 cells increased by FALEC over expression (Figure 5A, 5B). We further analyzed the protein levels of SHOC2 and ERK1/2, the downstream activator protein by FALEC. Consistent with the above results, upregulation of FALEC increased the protein level of SHOC2 and p-ERK1/2, while upregulation of miR-20a-5p mimics or knockdown SHOC2 significantly reduced their protein levels (Figure 5C, 5D). All these results demonstrated that FALEC contributes to the 5-FU-resistance of CCA cell via decreasing miR-20-5p to increase SHOC2/ERK1/2 activation.

## DISCUSSION

The incidence of cholangiocarcinoma (CCA) is gradually increased, and the early symptoms are easy to be ignored and misdiagnosed. In the absence of large-scale screening and effective screening methods, majority of CCA patients cannot be diagnosed early, and most of them are found in the middle and late stage, which was difficult to treat, and caused heavy disease burden and poor prognosis. Early diagnosis is the decisive factor to improve the survival rate of CCA patients. In this study, we collected a total of 20 pathologically confirmed CCA tissues and adjacent non-tumor tissues to detect the level of FALEC. The results showed that compared with the adjacent non-tumor control, FALEC RNA level was significantly higher in the CCA tissue samples, and further the expression of FALEC was higher in the 5-fluorouracil (5-FU) resistant patients. This result was consistent with previous reports, that upregulated FALEC promote the progression of digestive system tumors [8, 21].

In recent years, many studies have shown that long non-coding RNA (lncRNA) was abnormally expressed in CCA tissues, blood and exosomes, which has potential application value in diagnosis, prognosis assessment, and targeted therapy of CCA [22]. LncRNA generally regulate the expression of specific miRNAs as competitive endogenous RNA, acting on target molecules downstream of miRNA [23]. LncRNA can participate in tumor-related signal cascades, and further promote or inhibit tumor development through transcriptional activation/inhibition, epigenetic regulation, nuclear remodeling, mRNA stabilization/degradation, and miRNA sponge function [24, 25]. To further investigate the mechanism of FALEC in CCA, we used two CCA cell lines to establish 5-FU resistant CCA cell lines QBC939-R, and Huh-28-R. Then, we used siRNA interference to knock down the expression of FALEC in CCA cells, and the results showed that the proliferation, migration, and invasion of the cells were inhibited, suggesting that FALEC plays a carcinogenic role in CCA cells. The mechanism action of lncRNA mainly include: (1) directly binding to DNA or transcription factors to achieve gene expression regulation at the transcriptional level; (2) targeting mRNA, miRNA, or proteins and regulating their activity, stability, and post-transcriptional effects; (3) interference with chromatin complexes inhibits or activates gene expression in an epigenetic manner [26–28]. Based on existing research evidence, FALEC mainly functions by regulating its targeted miRNA.

In this study, ENCORI online database [29] was used to predict and indicates the target gene of FALEC might be miR-20a-5p. Previous studies have shown that miR-20a-5p was downregulated in CCA tissues [30]. In agreement with this pooled miRNA microarray results, our study showed that the level of miR-20a-5p also downregulated, and its expression increased after FALEC was knocked down. Dual luciferase assay confirmed that FALEC could target to the 3’UTR of miR-20a-5p, and FALEC could negatively regulate the expression of miR-20a-5p. As non-coding genes, miRNA plays a role by regulating target genes in related pathways. Further online databases including ENCORI, Target Scan, miRDB and MirTarBase were used to predict that the target gene of miR-20a-5p might be SHOC2. SHOC2 gene was located on chromosome 10q25 [31], SHOC2 protein was also known as SUR-8 (Suppressor of RAS-8), which can form ternary complex with RAS and RAF-1 to activate downstream ERK1/2 activity and participate in regulating cell proliferation, differentiation and other processes [32, 33]. The involvement of Scaffold protein in the process of drug resistance may be related to efflux pump inhibition [34]. Whether SHOC2 participates in the process of 5-FU via this mechanism need further investigation. In malignant melanoma, SHOC2 mediates acquired resistance of tumor cells to Raf inhibitor Vemurafenib through continuous activation of N-RAS [35]. SHOC2 can also promote the expression of LGALS3BP (lectin galactoside-binding soluble 3 binding protein) and so on to control the effect of ERK1/2 pathway on cell movement and adhesion, and the high expression of such proteins in blood and tumor tissues was associated with poor prognosis [36]. Moreover, inhibiting ERK/MAPK attenuates 5-FU resistance in colorectal cancer cells [37]. Sulahian, R et al. [38] revealed that deletion of SHOC2 deficiency weakens the adaptive reactivation of MAPK signaling pathway induced by MEK inhibitors. The above studies suggest that SHOC2 plays an important role in the development and drug resistance of tumors. We found SHOC2 mRNA level was negatively correlated with miR-20a-5p in CCA tissues. In addition, FALEC enhances SHOC2 expression through competitive adsorption of miR-20a-5p and promotes 5-FU resistance in CCA cells by activating ERK1/2 signaling pathway.

In conclusion, this study preliminary confirmed that FALEC was abnormally highly expressed in human CCA tissues and patients with poor response to 5 FU. Down-regulation of FALEC can inhibit the proliferation, migration, and invasion of QBC939 and Huh-28 cells by regulating miR-20a-5p /SHOC2 axis *in vitro*. ERK1/2 signaling pathway was also involved in 5-FU resistance of CCA cells. FALEC was expected to be a potential therapeutic target and molecular marker for CCA, and the FALEC / miR-20a-5p / SHOC2 axis regulatory network may provide a potential novel therapeutic strategy for the treatment of CCA.
